# Disentangling the Intertwined Genetic Bases of Root and Shoot Growth in Arabidopsis

**DOI:** 10.1371/journal.pone.0032319

**Published:** 2012-02-24

**Authors:** Marie Bouteillé, Gaëlle Rolland, Crispulo Balsera, Olivier Loudet, Bertrand Muller

**Affiliations:** 1 INRA, Laboratoire d'Ecophysiologie des Plantes sous Stress Environnementaux, UMR759, INRA, Montpellier, France; 2 SupAgro, Laboratoire d'Ecophysiologie des Plantes sous Stress Environnementaux, UMR759, INRA, Montpellier, France; 3 INRA, UMR1318, Institut Jean-Pierre Bourgin, Versailles, France; 4 AgroParisTech, UMR1318, Institut Jean-Pierre Bourgin, Versailles, France; University of California, Davis, United States of America

## Abstract

Root growth and architecture are major components of plant nutrient and water use efficiencies and these traits are the matter of extensive genetic analysis in several crop species. Because root growth relies on exported assimilate from the shoot, and changes in assimilate supply are known to alter root architecture, we hypothesized (i) that the genetic bases of root growth could be intertwined with the genetic bases of shoot growth and (ii) that the link could be either positive, with alleles favouring shoot growth also favouring root growth, or negative, because of competition for assimilates. We tested these hypotheses using a quantitative genetics approach in the model species *Arabidopsis thaliana* and the Bay-0×Shahdara recombinant inbred lines population. In accordance with our hypothesis, root and shoot growth traits were strongly correlated and most root growth quantitative trait loci (QTLs) colocalized with shoot growth QTLs with positive alleles originating from either the same or the opposite parent. In order to identify regions that could be responsible for root growth independently of the shoot, we generated new variables either based on root to shoot ratios, residuals of root to shoot correlations or coordinates of principal component analysis. These variables showed high heritability allowing genetic analysis. They essentially all yielded similar results pointing towards two regions involved in the root – shoot balance. Using Heterogeneous Inbred Families (a kind of near-isogenic lines), we validated part of the QTLs present in these two regions for different traits. Our study thus highlights the difficulty of disentangling intertwined genetic bases of root and shoot growth and shows that this difficulty can be overcome by using simple statistical tools.

## Introduction

Roots receive an increasing attention in particular in the context of a changing agriculture and climate. Their development, growth and architecture are thought to be major components of plant nutrient and water use efficiencies [1]–[3]. Thus, they are proposed to be one of the leverage for the next green revolution [4]. Moreover, roots can substantially contribute directly or indirectly to carbon sequestration [5], making them key actors in global earth carbon budget. Interspecific and intraspecific variation for root growth and architecture have been repeatedly reported in various species or genera opening the door to the design and breeding of crop or varieties carrying most useful root features adapted to various environmental conditions [6]. Reports showing that such strategy can bring substantial improvement of plant fitness and production are now accumulating [7]–[9]. Moreover, not only root growth, but also the partitioning of biomass between the root and the shoot has been reported to be a key parameter related to plant growth rate, life habitats and responses to environmental constraints such as nutrient deficiencies, drought or light [10]–[11].

Genetic analysis leading to the identification of quantitative traits loci (QTL) responsible for the variation of root variables have been conducted in a variety of species, the earliest reports being on rice [12] to more recently tree species [13]. QTLs have thus been reported for total root biomass [12], [14], root length [15], root branching [16], [17], proportion of shallow *vs* deep roots [18] or root angle [19]. QTL analyses in the model species *Arabidopsis thaliana* have also been engaged in a variety of mapping populations [20]–[23]. Such studies have pointed towards QTLs involved in either constitutive traits related to root growth [24] or towards QTLs associated with root growth *responses* to the environment such as responses to low phosphate [25], [26], to low nitrogen [27], to water deficit [28], [29], or to osmotic stress [30], [31]. These distinct variables types (intrinsic and response) are thought to reflect the probable different nature of the molecular pathways involved [32]. Whether constitutive or environmentally determined, very few root QTLs have been conducted to cloning, all being in Arabidopsis [33]–[35].

Among most genetic studies published so far on the determinism of root system architecture or dimension, the aerial part of the plant is sometimes [11], [29], [36], [37] but not always considered as a possible co-variable of the root variables [7], [17], [24]. However, roots, as sink organs, strongly rely on the continuous supply of assimilate from the shoot for both their growth and expansion, as well as for the establishment of their architecture. Indeed, changes in shoot biomass, in shoot growth rate or in intercepted irradiance can deeply modify root growth and architecture [38]–[40]. Intuitively, an increased shoot growth could be expected to be either favourable or disadvantageous for root growth. Favourable because a higher shoot growth is expected to increase carbon capture and assimilate export to sinks and disadvantageous because higher shoot growth may yield to competition for assimilates. Arguments for both have been reported. Root elongation rate is decreased by shoot pruning that reduces the source of carbon [38]. Similarly, root elongation rate is increased by increasing irradiance [39], [41] in association with higher sugar content in the root [40]. By contrast, in some species, flushes of shoot growth strongly impair root growth when they occur [42] probably as a result of competition for assimilates [43]. Probably because roots represent a high C cost, selection for high yielding varieties has been accompanied by a reduction of biomass partitioning towards roots [44], [45]. Both types of links may contribute to the co-ordination of root and shoot growth in response to challenging external conditions [10], [46]. This co-ordination has been shown to take place within a narrow range when a large spectrum of species is considered [47].

From these informations, we hypothesized that at least part of the genetic variation for root growth could be related to genetic variation for shoot growth. In this study, we tested this hypothesis using a quantitative genetics approach in the model species *Arabidopsis thaliana* and the Bay-0×Shahdara recombinant inbred line population [20]. In order to have easy access to the root system, all experiments were performed in hydroponics.

## Materials and Methods

### Genetic material

Two sets of genotypes were used. The first one is a sub-population of 165 Recombinant Inbred Lines (RILs) from the Bay-0×Shahdara RIL population [20], chosen to capture maximum recombination. This population is genotyped with 69 microsatellites markers. Complete genetic and phenotypic information on this population is available at http://dbsgap.versailles.inra.fr/vnat/Documentation/33/DOC.html. A second set of genotypes was used for QTL validation. Heterogeneous Inbred Families (HIF) lines were derived from residual heterozygosity remaining in some of the F6 RIL at markers of interest [24]. For each of these lines, 20 plants were individually genotyped at the segregating markers and two homozygous plants for each of the parental alleles (Bay or Sha) were selected and selfed to produce seeds for further phenotypic analysis. For each QTL to be validated, 2 independent HIFs (HIF083 and HIF107 at the bottom of chr. 1 and HIF338 and HIF004 at the top of chr. 3) were available and used for phenotyping. Therefore, in the experiments concerning HIFs, for each four HIF (HIF083, HIF107, HIF338, HIF004) we compared the mean value of two “sisters” HIFs carrying Bay allele to two other HIFs carrying the Sha allele at the region of interest. All this material was obtained from Versailles Arabidopsis Stock Centre (http://dbsgap.versailles.inra.fr/vnat/).

### Plant growth conditions

Seeds were surface-sterilized for 15 minutes in a mixture of bleach in 50% (v/v) ethanol, rinsed once in ethanol and then 3 times in sterile water. Two seeds were laid down at the surface of small cones (bottom part of 0.5 ml Eppendorf cut at both ends) filled with nutritive media (agar 0.65% w/v+nutrient solution). Cones were stored in Petri plates at 4°C in darkness during 24 hours. Petri plates were installed in the growth chamber for 5 days to allow seed germination. No difference in terms of time for germination was detected between the different lines. Then, cones were transferred to the hydroponic system composed of 20×30 cm styrofoam plates (thickness 1.0 cm) pierced by 96 holes and adjusted to float on nutrient solution in 5 L containers. The solution (one-tenth-strength modified Hoagland solution) was renewed every 3 days. All experiments were performed in a set of identical, 1 m^2^ growth cabinets, under the following climate: temperature was kept constant at 21°C days and night, relative air humidity was set at 80% in order to reach an air vapor pressure deficit of 0.6 kPa, light was 180 µmol.m-2.s-1 provided by a mixture of sodium and HQI lamps, during a 12 h photoperiod. To avoid any unconsidered bias due to location within the growth cabinet, containers were randomly moved from one location to another every day.

### Experiments

During the first experiment, the 165 RIL of the Bay-0×Shahdara population and the two parental lines were grown. 15 cones were used per RIL from which 8 homogeneous plants were selected 12 days after sowing. Four plants randomly selected among the 8 were then harvested at two dates (20 and 24 days after sowing) in order to evaluate the robustness of the results. The second date was chosen to avoid any overlap between plants and to avoid any interaction with flowering. Indeed, at that time, none of the RILs displayed visible floral bud. The 15 cones of every RIL were shared out in three different containers to avoid possible block effect. To lighten the daily work load, experiment 1 was performed as 3 waves of sowing spaced by 3 days with 55–56 RIL at each date. Three successive experiments were dedicated to the culture of four HIFs. On average, 80 plants of each HIF were cultivated, and at least 12 homogeneous plants per line were selected 2 weeks after sowing for the harvests which were then performed 20 and 24 days after sowing.

### Variables measurement and data acquisition

At each harvest, all the replicate plants of each genotype were gently removed from the hydroponic system, and their shoot and root parts were separated. None of the plants were at the bolting stage so shoot samples comprised vegetative parts only. Each leaf blades of the rosette was detached from the petioles, spread out and stuck with double-sided adhesive on a sheet of paper. Total leaf area was determined as the sum of the areas of each leaf blade. Blades were then gathered with petioles for estimation of shoot dry weight following 2 days at 80°C. In order to capture root architecture, root systems were gently spread at the surface of large (20×20 cm) Petri plates filled with water and a numerical image was taken at 800 dpi using a scanner in transmission mode. Images were later processed to measure primary and total root length using Image-J software and customized macros (Figure S1, available at http://bioweb.supagro.inra.fr/phenopsis/MacroImageJ.php). After image capture, root systems were individually stored into 96 well plates each containing pre-weighed aluminium cell-cup to facilitate weighing. The plates were then oven dried for 2 days at 80°C and cups were weighed using a 5 digits balance to measure root dry weight.

### QTL detection and statistical analysis

All statistical analyses were performed using the computer package SPSS 11.0.1 for Windows (SPSS) and the R software. Statistical differences between HIF lines were tested by t-test. Correlations were analysed using Pearson statistics. Normality of the distributions of each variable among the lines was verified by Shapiro test. Heritability (broad sense) was estimated as the proportion of variance explained by between-line differences based on measurements of four plants per line, at each date of harvest. A first QTL detection using simple interval mapping (IM) was performed with the MapQTL5 software (MAPQTL®5, Kyazma BV, Wageningen, the Netherlands). Cofactors were then selected using the ‘automatic cofactor selection’ chromosome per chromosome, and were used for Composite Interval Mapping. The cofactors for which no QTL were detected (LOD score under a 95% LOD threshold (LOD<2.4) estimated by permutation tests implemented in MapQTL5 using at least 1,000 permutations of the original dataset) were removed. The Epistat software [48] was used to identify possible epistatic interactions between markers. Then, these epistatic interactions were tested using the GLM of the statistical package of SPSS 11.0.1 for Windows. QTL models combining main effect QTLs and epistatic QTLs were statistically tested. The estimated additive effect, the percentage of variance explained by each individual QTL, and the total variance explained by the QTL model were obtained using the same package.

## Results

### Tight correlations between root and shoot growth variables in RILs

**Figure 1:**
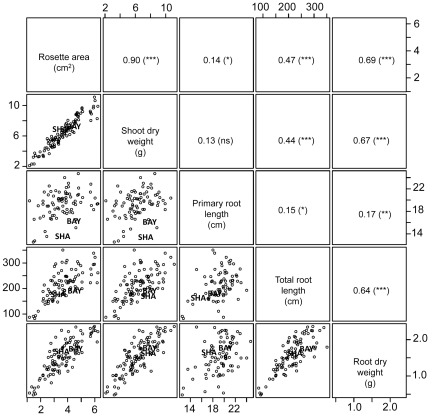
Correlation matrix between the different root and shoot growth variables at 24 days after sowing within the 165 individuals of the Bay-0×Shahdara RIL population. Dots represent the mean values of each RIL (4 individuals), and Bay-0 and Shahdara parental lines are indicated. Pearson's coefficients (r) associated to correlations are shown with their p-value (***, p-value<0.001, **, p-value<0.01, *, p-value<0.05, ns, p-value>0.05). Shoot and root dry weight are expressed in mg, rosette area in cm^2^, total and primary root length in cm.

A large variability among the RILs was observed for each of the 5 variables with ample transgression from the parents (Figure 1 and Figure S2A). At 24 days after sowing (Figure 1), shoot dry weight varied from 2 to 10 mg, root dry weight varied from 0.5 to 2 mg while primary root length varied between 12 and 24 cm. A similar range of variation was observed at 20 days after sowing (Figure S2A).

Except for the correlation between shoot dry weight and primary root length, all shoot and root variables were significantly and positively correlated one to another (Figure 1), with Pearson's coefficients ranging from 0.14 to 0.90, 24 days after sowing (0.29 to 0.89 at 20 days after sowing, Figure S2A). Strong correlations were observed in all cases between shoot dry weight and rosette area, indicative of a limited variation of the specific leaf area. Correlations between shoot and root variables were the tightest with root dry weight, slightly less tight with total root length and much weaker with primary root length. Correlations between shoot variables and both root dry weight and total root length were of the same strength at both dates whereas the strength of the correlations between shoot variables and primary root length strongly decreased between 20 and 24 days after sowing (Figure S2B). Correlations were strong between root dry weight and total root length and much weaker between these variables and primary root length. The ranking of the correlations based on their strength was essentially maintained at both dates (Figure S2B).

### Correlations between shoot and root growth translated at the genetic level with common QTLs

**Figure 2:**
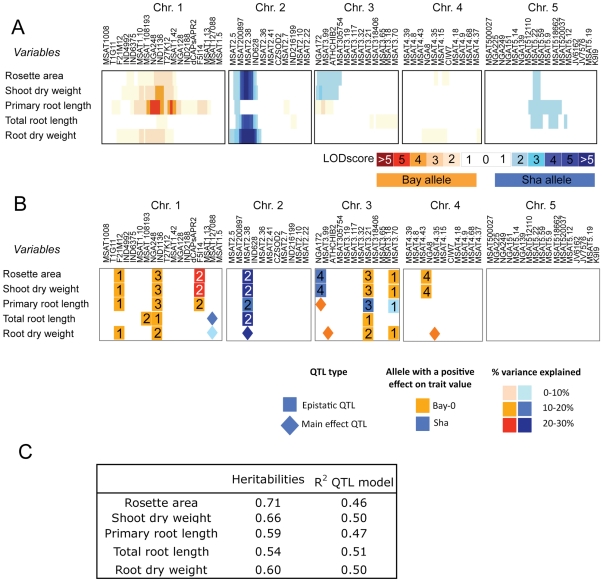
Genetic map of the QTL detected in the Bay-0×Shahdara for shoot and root growth variables. **A**. Map of the LOD score values all along the genome using Interval Mapping analysis. A color code indicates the parental allele which increases the value of the variables at the marker (blue for Sha alleles, and red for Bay alleles). The LOD score value is shown as different color intensities. **B**. Map of the regions involved in models combining main effects and epistatic QTLs. A color code indicates both the allele which increases the value of the variable at one specific region and the percentage of variance explained by the QTL. Identical numbers are indicated in the two partners of the epistatic interaction. **C**. Broad-sense heritability and r^2^ of the QTL models shown in B. Data are those obtained 24 days after sowing (the map at 20 days after sowing is shown as supplementary material).

Broad-sense heritability of root and shoot growth variables was high, ranging from 0.54 to 0.77, slightly higher for shoot than for root variables, and for the first date of harvest as compared to the second (Figure 2C at 24 days after sowing and Figure S3C at 20 days after sowing). A first detection of genomic regions involved in the control of these variables was performed using Interval Mapping (Figure 2A). However, despite the high heritability recorded for these variables, only few regions showing significant QTL (i.e. with LOD>2.4) were detected. Two regions showed a significant effect on almost all variables. The most important region was located at the top of chromosome 2, with Sha alleles contributing positively to the variables with LOD score ranging from 3 to 5 for all variables except for primary root length. The middle of chromosome 1 was also important, with Bay alleles contributing positively to all variables but total root length with the strongest effect on primary root length. The middle of chromosome 5 was also involved in shoot and root variables with positive effects of Sha alleles. The top of chromosome 3 was involved for shoot variables only. Both the top of chromosome 2 and the middle of chromosome 5 were strongly involved in all roots and shoot variables at 20 days after sowing (Figure S3A). This first analysis thus revealed some similarities of LOD profiles for shoot variables, root variables, but also between shoot and root variables.

An analysis was performed to identify possible epistatic interactions between markers. These epistatic interactions were individually tested before they were included in a global model gathering epistatic and main effect QTLs (Figure 2B and Table S1). The percentage of variance explained by the QTL models accounted for 46 to 51% of the phenotypic variance (Figure 2C), that corresponded to 60 to 90% of the genetic variance. This percentage was slightly higher at 24 days than at 20 days after sowing (Figure 2C, and Figure S3C), but similar markers were involved at both dates (Figure 2B and S3B). For all variables, genetic models were supported by both main effect QTLs and epistatic interactions involving interactors in the five chromosomes. As for the interval mapping analysis, shoot and root growth variables were determined by similar genomic regions (Figure 2B and S3B). The first epistasis (squares numbered 1 on Figure 2B) between the top of chromosome 1 (F21M12 marker) and the bottom of chromosome 3 (MSAT3.70) explained between 10 and 20% of total variance of all root and shoot variables, with positive effect of Bay allele at both loci except for primary root length at MSAT3.70. The second epistasis (squares #2) involved the bottom of chromosome 1 (F5I14, positive effect of Bay allele) and the top of chromosome 2 (MSAT2.38, positive effect of Sha allele), and explained 15 to 21% of the variance of all shoot variables as well as primary root length. A third epistasis was essentially associated with the middle of chromosome 1 (IND1136, positive effect of Bay allele) and the middle of chromosome 3 (MSAT3.21, positive effect of Bay alleles except for primary root length). Finally, an interaction between the top of chromosome 3 (squares #4, NGA172, positive effect of Sha allele) and the top of chromosome 4 (NGA8, positive effect of Bay allele), explained 10–15% of each variable, with an effect on shoot variables only. Interestingly, the region at the top of chromosome 3 also contained main effect QTLs controlling root variables with a positive effect of Bay allele. Analysis at 20 days after sowing (Figure S3B) pointed to essentially the same regions except that the third epistasis was not present. Even considering epistatic QTL models, very few QTLs specific for root growth variables were detected either at 24 days after sowing, or at 20 days after sowing (Figure S3B). Noteworthy, in many of the regions harbouring common QTLs for root and shoot variables, the same parental allele affected positively root and shoot variables except in the case of the bottom of chromosome 1 (although root and shoot QTL peaks were separated by 2 markers) and the top of chromosome 3, parental alleles had opposite effects on shoot and root variables. This feature was visible at both dates of harvest (Figure S3B).

### Uncoupling root and shoot variables

**Figure 3:**
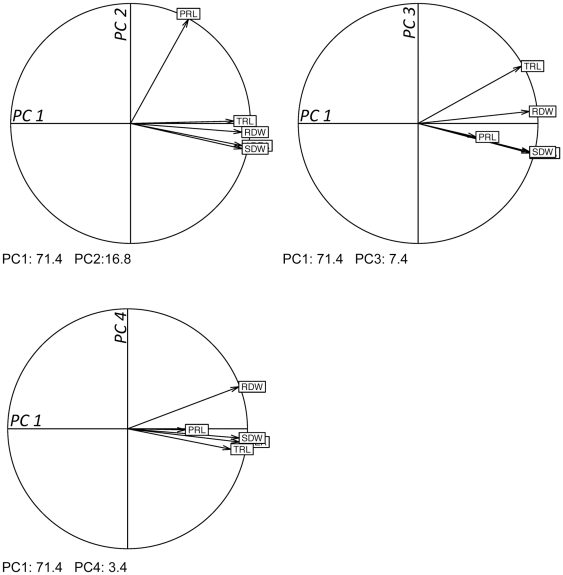
Principal component analysis based on root and shoot growth variables. As indicated at the bottom left of each circle, the first component (PC1) gathers 71.4% of the total variance whereas PC2, PC3 and PC4 gather 16.8, 7.4 and 3.4% of the total variance, respectively. The positions of the different variables, Rosette area (AREA), Shoot dry weight (SDW), total root length (TRL), primary root length (PRL), root dry weight (RDW) are represented. Data are those obtained 24 days after sowing.

**Figure 4:**
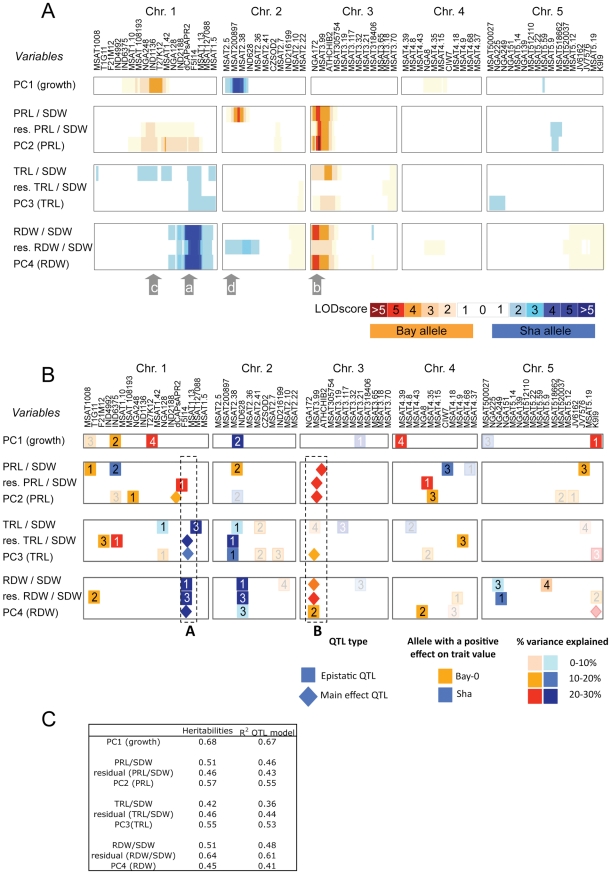
Genetic map of the QTLs detected for root to shoot ratio, residuals of correlations between root variables and shoot dry weight and coordinates in the principal component analysis. **A**. Map of the LOD score values all along the genome using Interval Mapping analysis. A color code indicates the parental allele which increases the value of the variables at the marker (blue for Sha alleles, and red for Bay alleles). The LOD score value is shown as different color intensities. Arrows *a* to *d* refer to regions described in the text. **B**. Map of the regions involved in models combining main effects and epistatic QTLs. A color code indicates both the allele which increases the value of the variable at one specific region and the percentage of variance explained by the QTL. Identical numbers are indicated in the two partners of the epistatic interaction. A and B rectangles refer to regions controlling root related variable but not involved in global plant growth. Data are those obtained 24 days after sowing (the map at 20 days after sowing is shown as supplementary material). QTLs not retrieved in the map from 20 days after sowing plants are shown with a translucent color. **C**. Broad-sense heritability and r^2^ of the QTL models shown in B.

In order to disentangle the intertwined genetic bases of root and shoot growth, three sets of variables were calculated. First, a principal component analysis (PCA) was performed using all five shoot and root variables on the whole RIL dataset at 24 days after sowing (Figure 3). The first principal component (PC1) captured most of the inertia of the data (71% of total variance) and was strongly related to all variables but primary root length. This component was thus considered as accounting for whole plant growth. The second one (PC2) explained 16.8% of the variance of the population, and was mainly driven by the primary root length that accounted for 66% of the variation of this PC. The third principal component (PC3) accounted for 7.4% of the total variance and was mainly driven by total root length (that accounted for 44% of the variation along this component). Finally, principal component 4 (PC4) accounted for only 3.4% of the total variance and was mainly accounted for by root dry weight (accounting for 37% of the variation). Four additional variables were thus calculated as the coordinates of each RIL on these 4 PC. The same analysis was performed with data at 20 days after sowing (not shown). Second, for each RIL, the orthogonal residuals of the correlations (Figure 1) between root variables (root dry weight, total root length, and primary root length) and shoot dry weight were calculated. These residuals are indicative of the deviation of root (or shoot) growth from the main trend linking both variables. RILs located above the main trend were thus having relatively higher root growth and lower shoot growth than the average trend. Finally, ratios of root variables (root dry weight, total root length or primary root length) to shoot dry weight were calculated. These calculated variables all displayed medium to high heritability ranging from 0.42 to 0.68 (Figure 4C and Figure S4C).

### Identification of QTLs involved in the root-shoot balance

QTL detection was performed on residuals of root to shoot correlations, PC coordinates and root to shoot ratios. A first detection was performed using Interval Mapping (24 days after sowing, Figure 4A, and 20 days after sowing, Figure S4A). Four main regions were identified in this analysis (arrows a to d). The first principal component, corresponding to whole plant growth was mainly controlled by the middle of the first chromosome (arrow c, MSAT1.42) and by the top of chromosome 2 (arrow d, MSAT2.38). Among the root variables, only those related to primary root length showed association with these two QTLs controlling plant growth, with the same positive effect of Bay allele in c region, and opposite allelic effects in d region. Variables related to primary root length (PC2, residual of the correlation between primary root length and shoot dry weight, and the ratio between primary root length and shoot dry weight) were mainly controlled by the top of chromosome 3 (arrow b, AthCHIB2). This region was also associated with variables related to total root length and to root dry weight. For those variables related to total root length and root dry weight, another region at the bottom of chromosome 1 (a) was consistently involved. For the b region, a positive effect of the Bay allele was identified for all the root variables (primary root length, total root length, and root dry weight related variables). By contrast, the a region showed opposite allelic effect on either primary root length (positive effect of the Bay allele) or total root length and root dry weight (positive effect of Sha allele). Interestingly, among these four regions, the a and b regions were clearly detected at both 20 and 24 days while c and d regions were less clearly visible at 20 days after sowing (Figure S4A).

As with raw variables, several significant epistatic interactions (Table S2 and S3) were detected for each of these variables. QTLs models gathering main effect and epistatic QTLs individually explained 36 to 67% of total variance (Figure 4C and S4C). A major difference with the analysis from raw variables (Figure 2B) was that more regions, spread along the genome were involved, with some being involved in one specific variable, or at one date. Interestingly, we were able to detect some QTLs specific for plant growth (squares #4 in Figure 4B). Very few QTLs were associated with both whole plant growth and root related variables (eg MSAT2.38 and IND628 in Figure 4B and S4B) suggesting that our analysis was successful to separate these components from whole plant growth. Indeed, we detected several QTLs associated with root-shoot balance and/or root specific variables only with no overlap with PC1 associated regions either at 24 days after sowing, or at 20 days after sowing. This was particularly the case for two regions labelled ‘A’ and ‘B’ on Figure 4B. These regions were already detected on the QTL analysis using raw data but they were then associated with both root and shoot QTLs. Moreover, root QTLs in these regions were clearly reinforced with a higher density of main-effect QTLs accounting for a higher proportion of the variance. We therefore focused our attention on these two regions.

### Using Heterogeneous Inbred Families (HIF) to validate the role of A and B QTLs, specifically controlling root-shoot balance and/or root specific variables

**Figure 5:**
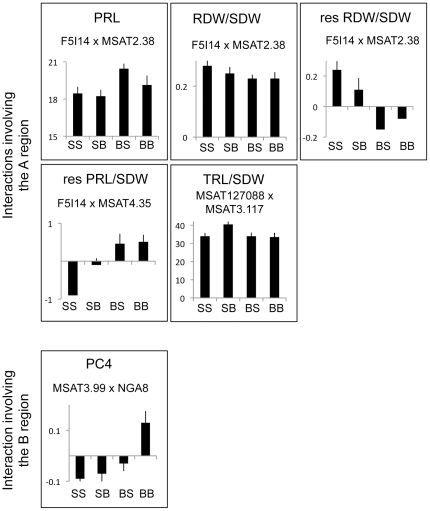
Mean values of root-related variables for the RILs in each of the four allelic classes for the 6 epistatic interactions involving the A and B regions: SS, SB, BS and BB refers to the RILs with the Sha allele at both markers indicated, the Sha allele at the first marker, and the Bay allele at the second, the Bay allele at the first marker, and the Sha allele at the second, and the Bay allele at both markers. Bars correspond to standard deviation.

**Figure 6:**
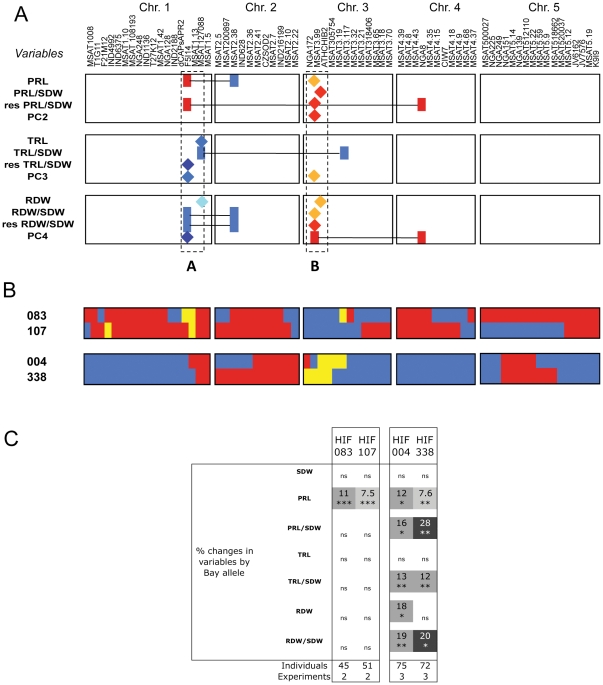
Validation of the role of A and B regions using Heterogeneous Inbred Families (HIF). **A.** Synthesis of the epistatic interactions involving either the bottom of chromosome 1 (A region) and/or the top of the chromosome 3 (B region) for root related variables. The markers involved in the interactions are colored in blue or red depending on the allele that increases the value of the trait, Sha or bay respectively. **B.** Genetic map of the RILs that were selfed to produce HIFs from residuals of heterozygosity in F6 generation of the RILs. Blue and red is for Sha and Bay alleles respectively. Yellow show heterozygous regions in RILs in F6. These regions are then fixed in the HIF progeny. **C.** Validation of the presence of two root QTLs on chromosome 1 and 3 using the four HIFs generated. The percentage of change of the variable induced by Bay allele compared to the Sha allele at the segregating region is indicated with the *t-test* p-value associated. Data shown gather harvests performed from 20 to 24 days after sowing. The number of individuals and experiments analyzed is indicated.

According to our analysis, ‘A’ and ‘B’ regions were involved in a total of 4 epistasis with other regions of the genome, among which one (F5I14×MSAT2.38) affected 3 different variables. The barplots representing the mean value of these variables for each allelic class are shown in Figure 5. The mean values of raw variables (primary root length, total root length, root dry weight and shoot dry weight) are indicated in Table S4. Region ‘A’ (F5I14-MSAT127088) was involved in an interaction with the top of chromosome 2 (MSAT2.38) to control 3 variables (primary root length, see Figure 2B, the root dry weight to shoot dry weight ratio and the residual of the correlation between root dry weight and shoot dry weight correlation, see Figure 4B). Region ‘A’ was also associated with MSAT4.35 to control the residual of the correlation between primary root length and shoot dry weight, and with MSAT3.117 to control the ratio between total root length and shoot dry weight. Region ‘B’ (MSAT3.99) was associated with NGA8 to control principal component 4. The main effect QTLs and the epistatic interactions involving the ‘A’ and ‘B’ regions are shown on the genetic map on Figure 6A.

In order to validate ‘A’ and ‘B’ main effect QTLs, we used a set of HIFs generated from residual heterozygosity detected at the F6 generation of the RIL. We therefore compared HIFs generated from the same RIL, but carrying either the Sha or Bay allele at the fixed region. Because the ‘A’ and ‘B’ QTLs were partly epistatic, we needed to consider the allele at the interacting loci (Figure 6B). We used two HIFs segregating at the ‘A’ region (HIF083 segregating at both F5I14 and MSAT1.13 and HIF107 segregating at MSAT1.13 only, Figure 6B) and two other HIFs segregating at the B region (HIF004 segregating from ATHCHIB2 to MSAT3.117 and HIF338 segregating from NGA172 to MSAT305754). Shoot dry weight, root dry weight, primary root length, total root length were measured in the different HIFs and ratios of root variables to shoot dry weight were computed (Figure 6C).

For both HIF083 and HIF107 (‘A’ region), a highly significant (p<0.001) positive effect of Bay allele on primary root length was found, that increased the variable by 7,5–11%. Although we cannot totally exclude that residual segregating regions in the genetic background of HIF083 and HIF107 (Figure 6) would interfere with the phenotype segregating at QTL ‘A’, we have paid attention that they would not be fixed together with the main segregating region in the different independent lines evaluated for each genotype (for example HIF083-Bay versus HIF083-Sha). Additionally, it is unlikely that HIF083 and HIF107 would segregate for the same phenotype if it was not because of their common heterozygous region. This confirmed the effect of the F5I14 locus on this variable (Figure 2). The F5I14 marker was often detected as interacting with MSAT2.38 but in both HIFs used, this second locus was fixed (Bay allele, Figure 6B). Therefore, we could not confirm that this QTL was epistatic. No other QTL was validated using HIF083 and HIF107 although QTLs had been detected at this region with raw variables (as main effect QTL) and with composite variables both as main effect and epistatic QTLs (Figures 2B and 4B). The epistatic interaction controlling the root dry weight to shoot dry weight ratio was not confirmed maybe because of the absence of the favourable allele (Sha) at the interactor MSAT2.38 in both HIF083 and HIF107 (Figure 6A and 6B).

The two HIFs used to validate the QTLs at the top of chromosome 3 originated from RILs displaying partly overlapping heterozygous regions with the HIF004 and HIF338 lines representing the most distal and proximal portion of the NGA172 – MSAT3.117 region respectively. Both lines validated the presence of QTLs in this region. Four traits were affected by variation in this region, the primary root length, the primary root length to shoot dry weight ratio, the root dry weight and the root dry weight to shoot dry weight ratio with a positive effect of Bay ranging from 7.6% (primary root length) to 28% (ratio between primary root length and shoot dry weight). No effect on total root length was confirmed in line with the lack of QTL for that trait in the region (Figure 2). The QTL responsible for shoot dry weight and rosette area variation at NGA172 was not confirmed using those lines. A reason could be that this marker interacts with NGA8 on chromosome 4, and the two HIF004 and HIF338 do not have the favourable allele (Bay) at this marker. Finally, the QTLs effects were almost similar for HIF004 and HIF338. A major difference between the two HIFs was the lack of confirmation of the QTL for root dry weight using the HIF338 which could indicate that the causal locus for this variable is located between MSAT305754 and MSTA3.19, a region that segregates only in HIF004. There was also a difference in the response of the two HIFs for the primary root length to shoot dry weight ratio, with a stronger effect of the QTL on HIF338.

## Discussion

### Coupling of root and shoot growth is translated at the genetic level

At the interspecific level, a strong coupling between root and shoot dimensions has been reported and conceptualised [47] suggesting that the diversification of biomass allocation strategies in plants has occurred within a narrow developmental window. At the intraspecific level, and in the absence of stress, a strong coupling between root and shoot growth have also been reported in wild species (*eg.* [49] in Hordeum, and [50] in Arabidopsis) as well as in crops (*eg.* [51] in maize, [52] in cotton). In our study, we found that the coupling between root and shoot growth still persisted when alleles originating from 2 parents were mixed up in a RIL population. The nature of this coupling is not known but it could be hydraulic, metabolic, hormonal or more probably a mixture of these. The absence of RILs with extreme root-shoot ratio could be explained by the strength of these coupling preventing the survival of RILs with extreme behaviour. Another explanation could be the overall multigenic and multilocus nature of the coupling between root and shoot growth traits that makes the occurrence of extreme individuals statistically unlikely. In line with these limitations, it would be useful to further explore the root-shoot relationships within inter-crossed mapping populations in which the number of alleles is increased [53], [54].

Root-shoot balance strongly depends on environmental conditions [10] with classically reported root-shoot ratio increases under low nutrient [55] or low water [56] or decreases under low light [51]. Moreover, genetic determinism of intrinsic and environmentally-related variation of root growth and architecture are likely to differ [32]. In our study, plants were grown in the absence of stress and results are thus likely to highlight intrinsic rules of root system development and biomass allocation. It will be highly relevant to evaluate how the strong root and shoot coupling found here withstand environmental variation.

Correlation between root and shoot variables were clearly translated at the genetic level with an essentially common set of main effect or epistatic QTL. Similar findings have been reported in previous reports in which roots and shoots parts were both considered. Some common QTL for shoot weight and root length, number and weight have been reported in maize [57] as well as in rice [29], [37]. In Arabidopsis grown under a range of N sources, some QTL overlapped between root and shoot dimensions or biomass [27]. In winter wheat, shoot and root biomass QTL were partly overlapping under the strong influence of the dwarfing gene *Rht*-B1 [36]. By contrast, some studies reported little or no correlation and no QTL overlap between shoot and root variables but these correspond most often to nutrient limiting situations. For instance, under low phosphate conditions, correlation between shoot dry weight and seminal root length in maize was moderate [58]. In Arabidopsis, low nitrate conditions led to a lack of common QTL between root and shoot variables [27]. In our work, the strength of the correlation and the degree of overlap between genetic models was higher than ever reported before. A possibility is that the hydroponic culture systems favoured a strong coupling between root and shoot growth because the liquid medium did not mechanically impede root growth as would a solid substrate.

### A large proportion of epistatic QTL

Very few of the regions identified with interval mapping analysis pointed to significant QTL. Models considering both main effect QTL and epistasic interactions were much more successful in accounting for the genetic variance of all variables measured. Epistasis is clearly acknowledged as being the rule when complex variables are considered [59], [60]. For instance, it has been suggested that epistatic effects are more important than additive effects for fitness traits [61], flowering time [62] and resistance to pathogens [63] and that these traits are best accounted for by a network of additive and epistatic effects [61]. In several instances, epistasis has been resolved at the gene level, including for growth-related traits [64]–[66]. Recently, a two-way epistasis was shown to be responsible for leaf growth maintenance under water deficit in Arabidopsis [67]. Our QTL analysis on raw variables highlighted several epistatic interactions in which the same interactors were involved in the control of root and shoot variables. These results reinforce the view of strongly intertwined genetic bases of root and shoot growth.

### Different degrees of overlap between genetic models associated with shoot and root variables are consistent with carbon partitioning rules governing root - shoot balance

An outcome of our study is that both the strength of correlations between root and shoot growth variables and the degree of overlap between shoot and root QTL models depended on the root variables considered. The weakest correlations and overlaps were found between shoot variables and primary root length. If we admit that root growth relies on exported assimilates from the shoot (*i.e.* the source in a source – sink terminology), our results suggest that primary root growth is under loose source limitation. This view fits well with recurrent findings that primary root elongation rate (by contrast with that of lateral roots) is unaltered when assimilate supply is modified [38], . Another noticeable feature of both the correlation and the QTL analysis is that the coupling between shoot and root was stronger with root dry weight than with total root length (see Figure 3). This is indicative that some genetic variation exists for the root length per mass ratio as already reported [39], [69]–[71]. This variable, classically called specific root length has been widely reported as a major trait relating root structure to function [72]. Moreover, variation of specific root length has also been reported to be triggered by variation in assimilate availability [73].

Together, these elements thus tend to associate the genetic coupling between root and shoot growth variables with the genetic determinism of assimilate partitioning. Such determinism could be associated with functional and/or structural variations in the sink itself (*e.g.* metabolic rates in root meristems or root meristem sizes, [41]), in the source part of plant (*e.g.* phloem loading rates or photosynthetic capacities) or along the path (*e.g.* sieve tube size or number, [74]). Ultimately, this variability would be associated with a variability in the rates of lateral root initiation and elongation as it is known that an increased C supply triggers these rates [68].

### Using composite variables to disentangle intertwined root and shoot variables and identify root QTLs

Multivariate analysis such as principle component analysis for genetic analysis has been used previously to simplify datasets and generate composite variables. For instance, it was used to identify QTL associated with complex variables such as leaf shape in Arabidopsis [75], in Brassica oleracea [76], or grain shape in wheat [77]. By contrast, in the present study, principle component analysis was used to extract components that show orthogonality to main trend, clearly driven by plant size. Principal components 2, 3 and 4 accounted for the root variation independently plant size. The coordinates along these axes, the ratios between root and shoot variables as well as the residuals of the correlations between root and shoot variables were jointly used to identify the genetic components that are not simply associated with whole plant growth.

Consistent with the initial assumption, the two main regions associated with PC1 were also the two main regions responsible for the control of all (root and shoot) raw variables with the same alleles favouring growth of organs. Moreover, the main QTL regions involved in the genetic control of root-related variables (bottom of chromosome 1 and top of chromosome 3) were independent of PC1 suggesting that our analysis successfully separated root and shoot component of plant growth. The role of these two ‘root-related’ regions was further examined by using HIF. The role of the first region was validated for primary root length determinism with the same allelic effect than in the QTL analysis but the HIFs did not validated the role of this region for other root-related variables. One explanation could be linked to the presence of two different QTLs in this region, the first one (confirmed) with a positive effect of Bay allele on primary root length, and the other with a positive effect of Sha, located maybe around MSAT127088, a region not segregating in the chosen HIFs. Another explanation could be the absence of the favourable allele at the interactor of the epistatis (MSAT2.38). The second region analysed yielded more straightforward results, possibly associated with several root-related variables. All these main effects QTLs were confirmed using HIFs, with a positive effect of Bay alleles in all cases. This is indicative of the presence of one single QTL having a pleiotropic effect on all these root-related variables. Except for the root dry weight, effects of the same amplitude were detected in both HIF004 and HIF338 suggesting they point to the overlapping hererozygous region between them as being responsible for the QTL (ATHCHIB2-MSAT305754).

The two regions highlighted co-localized with previously reported root QTLs. The QTL from lower half of chromosome 1 localized close to a root growth QTL previously identified as being due to a cell wall invertase gene [34]. It also co-localized with QTLs for both lateral root length and density in the same population, grown in Petri plates on agar media [24]. The top of chromosome 3 was previously shown to control lateral root length [24] as well as osmotic stress response of roots (Loudet O, unpublished data) and other growth related loci in another RIL set involving the Shahdara accession [21]. The conclusions of our study strengthen the role of these two regions as being responsible for root growth variables, independently of whole plant growth.

## Supporting Information

Figure S1 **Root length measurements using a macro developped on Image J by Volker Backer (Montpellier Rio Imaging), and available at** http://bioweb.supagro.inra.fr/phenopsis/MacroImageJ.php.
(TIF)

Figure S2 **A. Correlation matrix between the different root and shoot growth variables within the 165 individuals of the Bay-0×Shahdara RIL population.** Data are those obtained 20 days after sowing. Dots represent the mean values of each RIL (4 individuals), and Bay-0 and Shahdara parental lines are indicated. Pearson's coefficients (r) associated to correlations are shown with their p-value (***, p-value<0.001, **, p-value<0.01, *, p-value<0.05, ns, p-value>0.05). Shoot and root dry weight are expressed in mg, rosette area in cm^2^, total and primary root length in cm. **B.** Pearson coefficients for all correlations among the Bay-0×Shahdara RIL population at both 20 and 24 days after sowing (DAS).
(TIF)

Figure S3 **Genetic map of the QTL detected in the Bay-0×Shahdara for shoot and root growth variables.** Data are those obtained at 20 days after sowing. **A**. Map of the LOD score values all along the genome using Interval Mapping analysis. A color code indicates the parental allele which increases the value of the variables at the marker (blue for Sha alleles, and red for Bay alleles). The LOD score value is shown as different color intensities. **B**. Map of the regions involved in models combining main effects and epistatic QTLs. A color code indicates both the allele which increases the value of the variable at one specific region and the percentage of variance explained by the QTL. Identical numbers are indicated in the two partners of the epistatic interaction. **C**. Broad-sense heritability and r^2^ of the QTL models shown in B.
(TIF)

Figure S4 **Genetic map of the QTLs detected for root to shoot ratio, residuals of correlations between root variables and shoot dry weight and coordinates in the principal component analysis.** Data are those obtained 20 days after sowing. **A**. Map of the LOD score values all along the genome using Interval Mapping analysis. A color code indicates the parental allele which increases the value of the variables at the marker (blue for Sha alleles, and red for Bay alleles). The LOD score value is shown as different color intensities. Arrows *a* and *b* refer to regions described in the text. **B**. Map of the regions involved in models combining main effects and epistatic QTLs. A color code indicates both the allele which increases the value of the variable at one specific region and the percentage of variance explained by the QTL. Identical numbers are indicated in the two partners of the epistatic interaction. A and B rectangles refer to regions controlling root related variable but not involved in global plant growth. QTLs not retrieved in the map from 24 days after sowing plants are shown with a translucent color. **C**. Broad-sense heritability and r^2^ of the QTL models shown in B.
(TIF)

Table S1 **QTL models for the shoot and root growth variables.** AREA, SDW, PRL, TRL and RDW refer to rosette area, shoot dry weight, primary root length, total root length, and root dry weight respectively. Models are shown for both data at 20 and 24 days after sowing. The percentage of variance explained by the QTL model (R^2^ QTL model), the markers involved as main effect or epistasic, the p-value of the t-test, the percentage of variance explained by each term of the model, and the corresponding additive effect are indicated.
(TIF)

Table S2 **QTL models for three types of calculated variables**. Ratio between root variables (Root dry weight (RDW), Total root length (TRL), and Primary root length (PRL)) and Shoot dry weight (SDW), PCA coordinates on principal components 2, 3 and 4 (that are accounted for by primary root length, total root length and root dry weight respectively), and residuals of the correlations between root variables and shoot dry weight (SDW), at 20 days after sowing. The percentage of variance explained by the QTL model (R^2^ QTL model), the markers involved as main effect or epistasic, the p-value of the t-test, the percentage of variance explained by each term of the model, and the corresponding additive effect are indicated.
(TIF)

Table S3 **QTL models for three types of calculated variables**. Ratio between root variables (Root dry weight (RDW), Total root length (TRL), and Primary root length (PRL)) and Shoot dry weight (SDW), PCA coordinates on principal components 2, 3 and 4 (that are accounted for by primary root length, total root length and root dry weight respectively), and residuals of the correlations between root variables and shoot dry weight (SDW), at 24 days after sowing. The percentage of variance explained by the QTL model (R^2^ QTL model), the markers involved as main effect or epistasic, the p-value of the t-test, the percentage of variance explained by each term of the model, and the corresponding additive effect are indicated.
(TIF)

Table S4 **Mean (+/−SD) values of root and shoot variables for the RILs in each of the four allelic classes for the 4 epistatic interactions involving the A and B regions:** SS, SB, BS and BB refers to the RILs with the Sha or the Bay allele at the first and second marker respectively.
(TIF)

## References

[1] Ochoa IE, Blair MW, Lynch JP (2006). QTL analysis of adventitious root formation in common bean under contrasting phosphorus availability.. Crop Science.

[2] MacMillan K, Emrich K, Piepho H, Mullins C, Price A (2006). Assessing the importance of genotype×environment interaction for root traits in rice using a mapping population. I: a soil-filled box screen.. Theoretical and Applied Genetics.

[3] Manschadi AM, Hammer GL, Christopher JT, DeVoil P (2008). Genotypic variation in seedling root architectural traits and implications for drought adaptation in wheat (*Triticum aestivum* L.).. Plant and Soil.

[4] Lynch JP (2007). Roots of the second green revolution.. Australian Journal of Botany.

[5] Deyn GBD, Cornelissen JHC, Bardgett RD (2008). Plant functional traits and soil carbon sequestration in contrasting biomes.. Ecology Letters.

[6] de Dorlodot S, Forster B, Pagès L, Price A, Tuberosa R (2007). Root system architecture: opportunities and constraints for genetic improvement of crops.. Trends in Plant Science.

[7] Sanguineti MS, Li M, Maccaferri S, Corneti F, Rotondo (2007). Genetic dissection of seminal root architecture in elite durum wheat germplasm.. Annals of Applied Biology.

[8] Hochholdinger F, Tuberosa R (2009). Genetic and genomic dissection of maize root development and architecture.. Current Opinion in Plant Biology.

[9] Hammer GL, Dong Z, McLean G, Doherty A, Messina C (2009). Can changes in canopy and/or root system architecture explain historical maize yield trends in the U.S. corn belt?. Crop Science.

[10] Poorter H, Nagel O (2000). The role of biomass allocation in the growth response of plants to different levels of light, CO2, nutrients and water: a quantitative review.. Functional Plant Biology.

[11] Poorter H, Rijn CPE, Vanhala TK, Verhoeven KJF, Jong YEM (2005). A genetic analysis of relative growth rate and underlying components in Hordeum spontaneum.. Oecologia.

[12] Price AH, Tomos AD (1997). Genetic dissection of root growth in rice (Oryza sativa L.). II: mapping quantitative trait loci using molecular markers.. Theoretical and Applied Genetics.

[13] Kenis K, Keulemans J (2007). Study of tree architecture of apple (Malus×domestica Borkh.) by QTL analysis of growth traits.. Molecular Breeding.

[14] Reiter RS, Coors JG, Sussman MR, Gabelman WH (1991). Genetic analysis of tolerance to low-phosphorus stress in maize using restriction fragment length polymorphisms.. Theoretical and Applied Genetics.

[15] Zhang WP, Shen XY, Wu P, Hu B, Liao CY (2001). QTLs and epistasis for seminal root length under a different water supply in rice (*Oryza sativa* L.).. Theoretical and Applied Genetics.

[16] Robinson D (1986). Compensatory changes in the partitioning of dry matter in relation to nitrogen uptake and optimal variations in growth.. Annals of Botany.

[17] Chevalier F, Pata M, Nacry P, Doumas P, Rossignol M (2003). Effects of phosphate availability on the root system architecture: large-scale analysis of the natural variation between Arabidopsis accessions.. Plant, Cell & Environment.

[18] Liao H, Yan X, Rubio G, Beebe SE, Blair MW (2004). Genetic mapping of basal root gravitropism and phosphorus acquisition efficiency in common bean.. Functional Plant Biology.

[19] Giuliani S, Sanguineti MC, Tuberosa R, Bellotti M, Salvi S (2005). Root-ABA1, a major constitutive QTL, affects maize root architecture and leaf ABA concentration at different water regimes.. Journal of Experimental Botany.

[20] Loudet O, Chaillou S, Camilleri C, Bouchez D, Daniel-Vedele F (2002). Bay-0×Shahdara recombinant inbred line population: a powerful tool for the genetic dissection of complex traits in Arabidopsis.. Theoretical and Applied Genetics.

[21] El-Lithy ME, Clerkx EJ, Ruys GJ, Koornneef M, Vreugdenhil D (2004). Quantitative trait locus analysis of growth-related traits in a new Arabidopsis recombinant inbred population.. Plant Physiology.

[22] Balasubramanian S, Schwartz C, Singh A, Warthmann N, Kim MC (2009). QTL mapping in new arabidopsis thaliana advanced intercross-recombinant inbred lines.. PLoS ONE.

[23] Kover PX, Valdar W, Trakalo J, Scarcelli N, Ehrenreich IM (2009). A Multiparent Advanced Generation Inter-Cross to fine-map quantitative traits in Arabidopsis thaliana.. PLoS Genet.

[24] Loudet O, Gaudon V, Trubuil A, Daniel-Vedele F (2005). Quantitative trait loci controlling root growth and architecture in Arabidopsis thaliana confirmed by heterogeneous inbred family.. Theoretical and Applied Genetics.

[25] Reymond M, Svistoonoff S, Loudet O, Nussaume L, Desnos T (2006). Identification of QTL controlling root growth response to phosphate starvation in Arabidopsis thaliana.. Plant, Cell & Environment.

[26] Li J, Xie Y, Dai A, Liu L, Li Z (2009). Root and shoot traits responses to phosphorus deficiency and QTL analysis at seedling stage using introgression lines of rice.. Journal of Genetics and Genomics.

[27] Rauh B, Basten C, Buckler E (2002). Quantitative trait loci analysis of growth response to varying nitrogen sources in Arabidopsis thaliana.. Theoretical and Applied Genetics.

[28] Courtois B, McLaren G, Sinha PK, Prasad K, Yadav R (2000). Mapping QTLs associated with drought avoidance in upland rice.. Molecular breeding.

[29] Cui K, Huang J, Xing Y, Yu S, Xu C (2008). Mapping QTLs for seedling characteristics under different water supply conditions in rice (*Oryza sativa*).. Physiologia Plantarum.

[30] Xiong L, Wang RG, Mao G, Koczan JM (2006). Identification of drought tolerance determinants by genetic analysis of root response to drought stress and abscisic acid.. Plant physiology.

[31] Gerald FJN, Lehti-Shiu MD, Ingram PA, Deak KI, Biesiada T (2006). Identification of quantitative trait loci that regulate Arabidopsis root system size and plasticity.. Genetics.

[32] Malamy J (2005). Intrinsic and environmental response pathways that regulate root system architecture.. Plant, Cell & Environment.

[33] Mouchel CF, Briggs GC, Hardtke CS (2004). Natural genetic variation in Arabidopsis identifies BREVIS RADIX, a novel regulator of cell proliferation and elongation in the root.. Genes and Development.

[34] Sergeeva LI, Keurentjes JJB, Bentsink L, Vonk J, van der Plas LHW (2006). Vacuolar invertase regulates elongation of Arabidopsis thaliana roots as revealed by QTL and mutant analysis.. Proceedings of the National Academy of Sciences of the United States of America.

[35] Svistoonoff S, Creff A, Reymond M, Sigoillot-Claude C, Ricaud L (2007). Root tip contact with low-phosphate media reprograms plant root architecture.. Nature Genetics.

[36] Laperche A, Devienne-Barret F, Maury O, Le Gouis J, Ney B (2006). A simplified conceptual model of carbon/nitrogen functioning for QTL analysis of winter wheat adaptation to nitrogen deficiency.. Theoretical and Applied Genetics.

[37] Kamoshita A, Wade L, Ali M, Pathan M, Zhang J (2002). Mapping QTLs for root morphology of a rice population adapted to rainfed lowland conditions.. Theoretical and Applied Genetics.

[38] Farrar JF, Jones CL (1986). Modification of respiration and carbohydrate status of barley roots by selective pruning.. New Phytologist.

[39] Aguirrezabal LAN, Deleens E, Tardieu F (1994). Root elongation rate is accounted for by intercepted PPFD and source-sink relations in field and laboratory-grown sunflower.. Plant, Cell and Environment.

[40] Freixes S, Thibaud M, Tardieu F, Muller B (2002). Root elongation and branching is related to local hexose concentration in Arabidopsis thaliana seedlings.. Plant, Cell & Environment.

[41] Muller B, Stosser M, Tardieu F (1998). Spatial distributions of tissue expansion and cell division rates are related to irradiance and to sugar content in the growing zone of maize roots.. Plant, Cell & Environment.

[42] Thaler P, Pagès L (1996). Root apical diameter and root elongation rate of rubber seedlings (*Hevea brasiliensis*) show parallel responses to photoassimilate availability.. Physiologia Plantarum.

[43] Thaler P, Pagès L (1998). Modelling the influence of assimilate availability on root growth and architecture.. Plant and Soil.

[44] Bruce WB, Edmeades GO, Barker TC (2003). Molecular and physiological approaches to maize improvement for drought tolerance.. Journal of Experimental Botany,.

[45] Siddique KHM, Belford RK, Tennant D (1990). Root∶shoot ratios of old and modern, tall and semi-dwarf wheats in a mediterranean environment.. Plant and soil,.

[46] Brouwer R (1962). Distribution of dry matter in plants.. Netherlands Journal of Agricultural Science.

[47] Enquist BJ, Niklas KJ (2002). Global Allocation Rules for Patterns of Biomass Partitioning in Seed Plants.. Science.

[48] Chase K, Adler FR, Lark KG (1997). Epistat: a computer program for identifying and testing interactions between pairs of quantitative trait loci.. Theoretical and Applied Genetics.

[49] Van Rijn CPE, Heersche I, Van Berkel YEM, Nevo E, Lambers H, Poorter H (2000). Growth characteristics in Hordeum spontaneum populations from different habitats.. New Phytologist.

[50] Li B, Suzuki JI, Hara T (1998). Latitudinal variation in plant size and relative growth rate in Arabidopsis thaliana.. Oecologia.

[51] Hebert Y, Guingo E, Loudet O (2001). The response of root/shoot partitioning and root morphology to light reduction in maize genotypes.. Crop science.

[52] McMichael BL, Quisenberry JE (1991). Genetic variation for root-shoot relationships among cotton germplasm.. Environmental and experimental botany.

[53] Kover PX, Valdar W, Trakalo J, Scarcelli N, Ehrenreich IM, Purugganan MD, Durrant C, Mott R (2009). A Multiparent Advanced Generation Inter-Cross to fine-map quantitative traits in Arabidopsis thaliana.. PLoS Genet.

[54] Huang X, Paulo MJ, Boer M, Effgen S, Keizer P (2011). Analysis of natural allelic variation in Arabidopsis using a multiparent recombinant inbred line population.. Proceedings of the National Academy of Sciences of the United states of America.

[55] McConnaughay KDM, Coleman JS (1998). Can plants track changes in nutrient availability via changes in biomass partitioning?. Plant and soil.

[56] Hummel I, Pantin F, Sulpice R, Piques M, Rolland G (2010). Arabidopsis plants acclimate to water deficit at low cost through changes of carbon usage: an integrated perspective using growth, metabolite, enzyme, and gene expression analysis.. Plant Physiology.

[57] Hund A, Fracheboud Y, Soldati A, Frascaroli E, Salvi S (2004). QTL controlling root and shoot traits of maize seedlings under cold stress.. Theoretical and Applied Genetics.

[58] Zhu J, Mickelson S, Kaeppler S, Lynch J (2006). Detection of quantitative trait loci for seminal root traits in maize (*Zea mays* L.) seedlings grown under differential phosphorus levels.. Theoretical and Applied Genetics.

[59] Carlborg O, Haley CS (2004). Epistasis: too often neglected in complex trait studies?. Nature Review Genetics.

[60] Cooper M, van Eeuwijk FA, Hammer GL, Podlich DW, Messina C (2009). Modelling QTL for complex traits: detection and context for plant breeding.. Current Opinion in Plant Biology.

[61] Malmberg RL (2005). Epistasis for Fitness-Related Quantitative Traits in Arabidopsis thaliana Grown in the Field and in the Greenhouse.. Genetics.

[62] Juenger T, Sen S, Stowe K, Simms E (2005). Epistasis and genotype-environment interaction for quantitative trait loci affecting flowering time in Arabidopsis thaliana.. Genetica.

[63] Rowe HC, Kliebenstein DJ (2008). Complex genetics control natural variation in Arabidopsis thaliana resistance to *Botrytis cinerea*.. Genetics.

[64] Kroymann J, Mitchell-Olds T (2005). Epistasis and balanced polymorphism influencing complex trait variation.. Nature.

[65] Bikard D, Patel D, Le Metté C, Giorgi V, Camilleri C (2009). Divergent evolution of duplicate genes leads to genetic incompatibilities within A. thaliana.. Science.

[66] Vlad D, Rappaport F, Simon M, Loudet O (2010). Gene transposition causing natural variation for growth in Arabidopsis thaliana.. PloS Genetics.

[67] Tisné S, Schmalenbach I, Reymond M, Dauzat M, Pervent M (2010). Keep on growing under drought: genetic and developmental bases of the response of rosette area using a recombinant inbred line population.. Plant, Cell & Environment.

[68] Bingham IJ, Stevenson EA (1993). Control of root growth: effects of carbohydrates on the extension, branching and rate of respiration of different fractions of wheat roots.. Physiologia Plantarum.

[69] Robinson D (1986). Compensatory changes in the partitioning of dry matter in relation to nitrogen uptake and optimal variations in growth.. Annals of Botany.

[70] Ryser P, Eek L (2000). Consequences of phenotypic plasticity vs. interspecific differences in leaf and root traits for acquisition of aboveground and belowground resources.. American Journal of Botany.

[71] Ostonen I, Püttsepp Ü, Biel C, Alberton O, Bakker MR (2007). Specific root length as an indicator of environmental change.. Plant Biosystems.

[72] Roumet C, Urselay C, Diaz S (2006). Suites of root traits differ between annual and perennial species growing in the field.. New phytologist.

[73] Guo DL, Mitchell J, Hendricks JJ (2004). Fine root branch orders respond differentially to carbon source-sink manipulations in a longleaf pine forest.. Oecologia.

[74] Wardlaw IF (1990). The control of carbon partitioning in plants.. New Phytologist.

[75] Langlade NB, Feng X, Dransfield T, Copsey L, Hanna AI (2005). Evolution through genetically controlled allometry space.. Proceedings of the National Academy of Sciences of the united states of america.

[76] Sebastian RL, Kearsey MJ, King GJ (2002). Identification of quantitative trait loci controlling developmental characteristics of *Brassica oleracea* L.. Theoretical and Applied Genetics.

[77] Iwata H, Ebana K, Uga Y, Hayashi T, Jannink J (2010). Genome-wide association study of grain shape variation among *Oryza sativa* L. germplasms based on elliptic Fourier analysis.. Molecular Breeding.

