# Supplementary figures and images for: Disentangling the Intertwined Genetic Bases of Root and Shoot Growth in Arabidopsis
Source: PLoS One. 2012 Feb 24;7(2):e32319. doi: 10.1371/journal.pone.0032319 (PMC3286473; doi:10.1371/journal.pone.0032319)

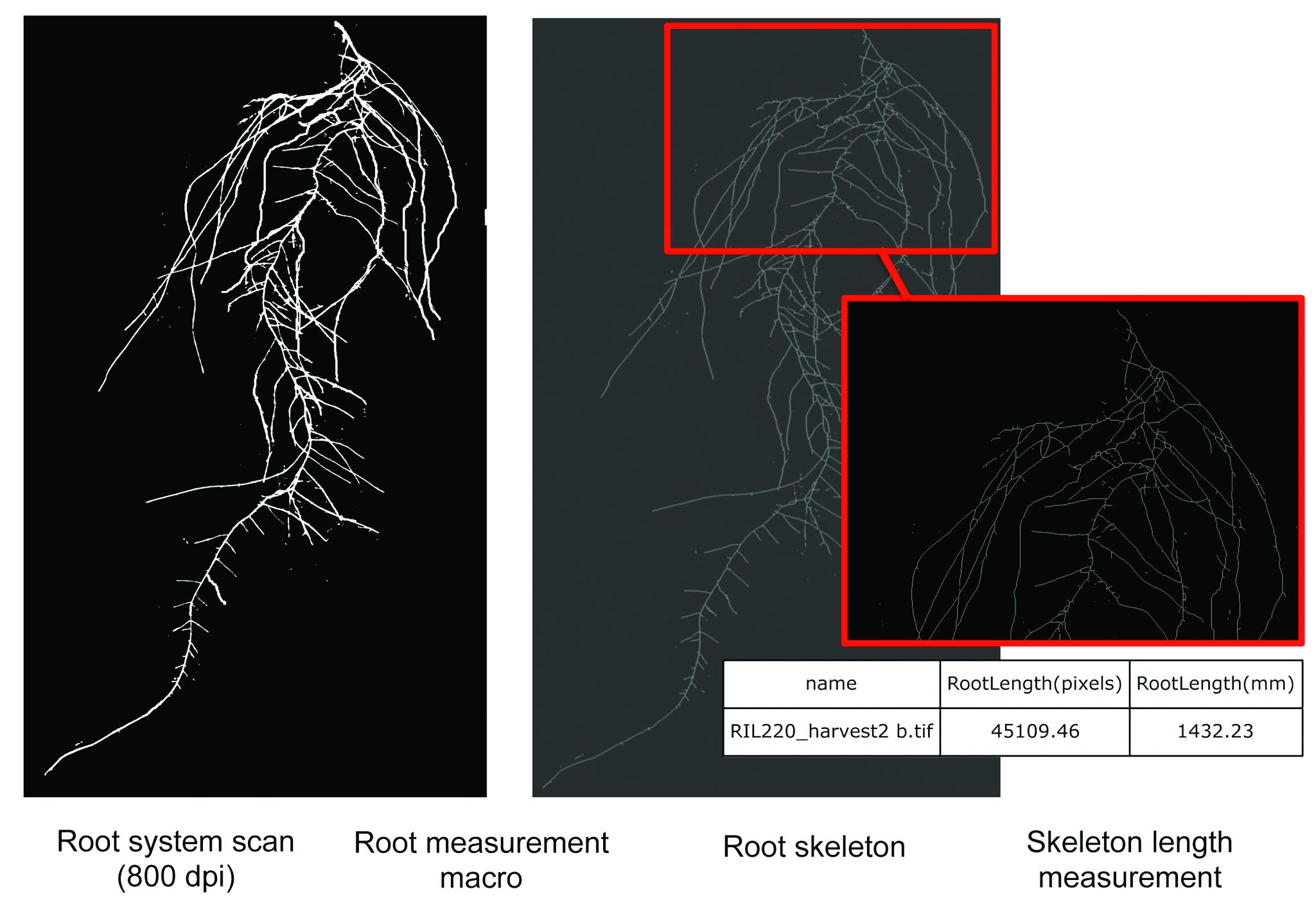

Supplement: Figure S1 — Root length measurements using a macro developped on Image J by Volker Backer (Montpellier Rio Imaging), and available at http://bioweb.supagro.inra.fr/phenopsis/MacroImageJ.php. (TIF) [file pone.0032319.s001.tif]

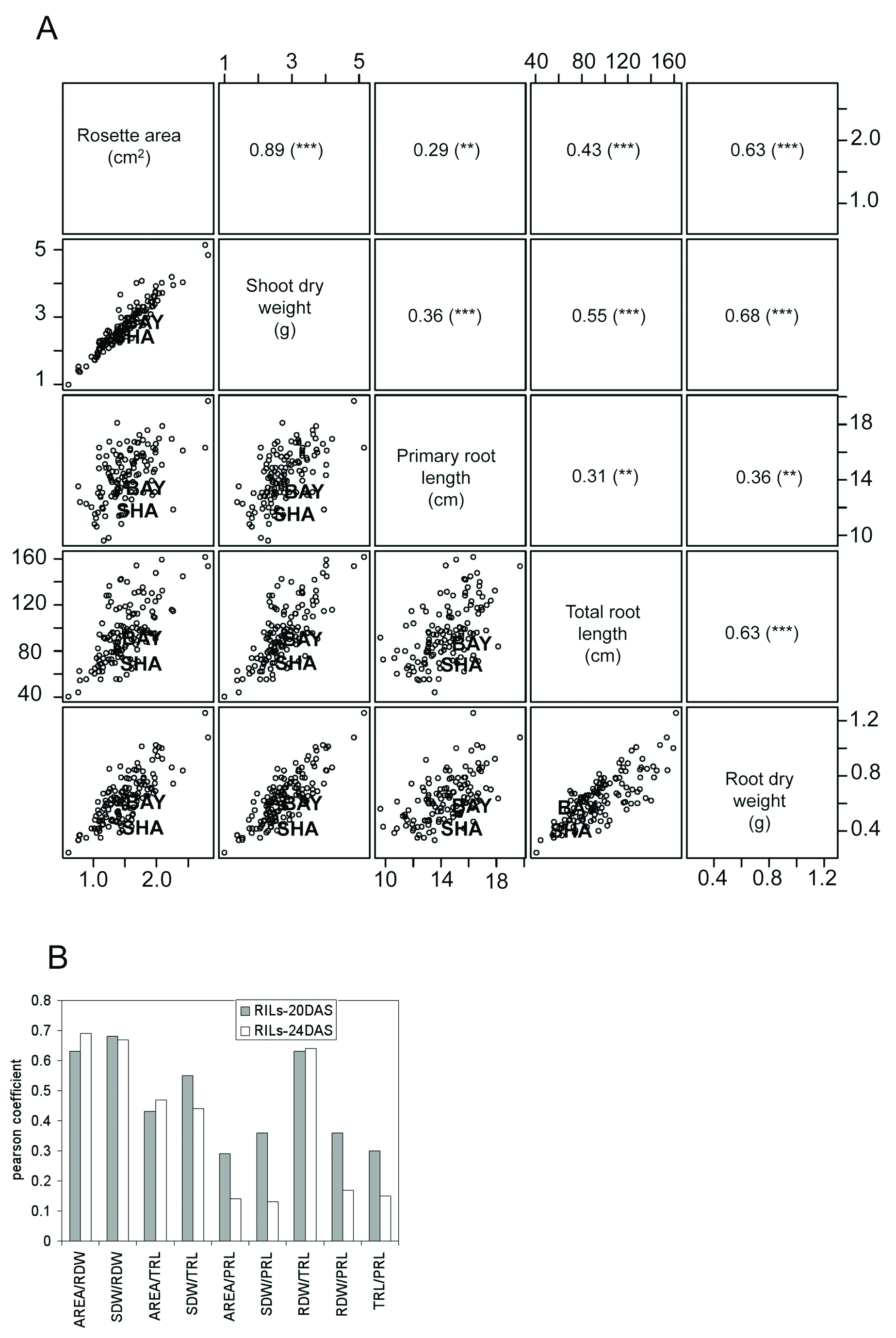

Supplement: Figure S2 — A. Correlation matrix between the different root and shoot growth variables within the 165 individuals of the Bay-0×Shahdara RIL population. Data are those obtained 20 days after sowing. Dots represent the mean values of each RIL (4 individuals), and Bay-0 and Shahdara parental lines are indicated. Pearson's coefficients (r) associated to correlations are shown with their p-value (***, p-value<0.001, **, p-value<0.01, *, p-value<0.05, ns, p-value>0.05). Shoot and root dry weight are expressed in mg, rosette area in cm2, total and primary root length in cm. B. Pearson coefficients for all correlations among the Bay-0×Shahdara RIL population at both 20 and 24 days after sowing (DAS). (TIF) [file pone.0032319.s002.tif]

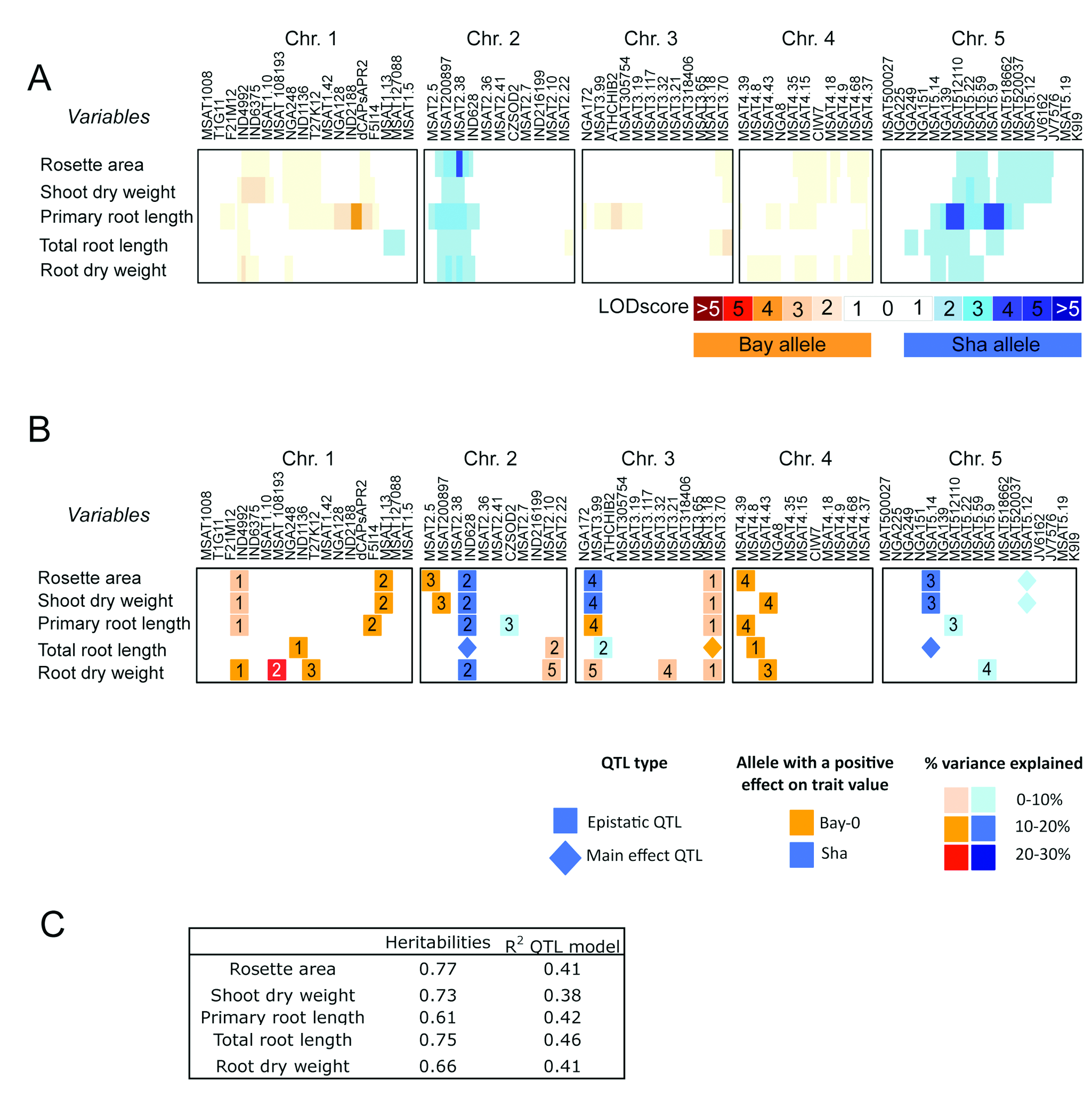

Supplement: Figure S3 — Genetic map of the QTL detected in the Bay-0×Shahdara for shoot and root growth variables. Data are those obtained at 20 days after sowing. A. Map of the LOD score values all along the genome using Interval Mapping analysis. A color code indicates the parental allele which increases the value of the variables at the marker (blue for Sha alleles, and red for Bay alleles). The LOD score value is shown as different color intensities. B. Map of the regions involved in models combining main effects and epistatic QTLs. A color code indicates both the allele which increases the value of the variable at one specific region and the percentage of variance explained by the QTL. Identical numbers are indicated in the two partners of the epistatic interaction. C. Broad-sense heritability and r2 of the QTL models shown in B. (TIF) [file pone.0032319.s003.tif]

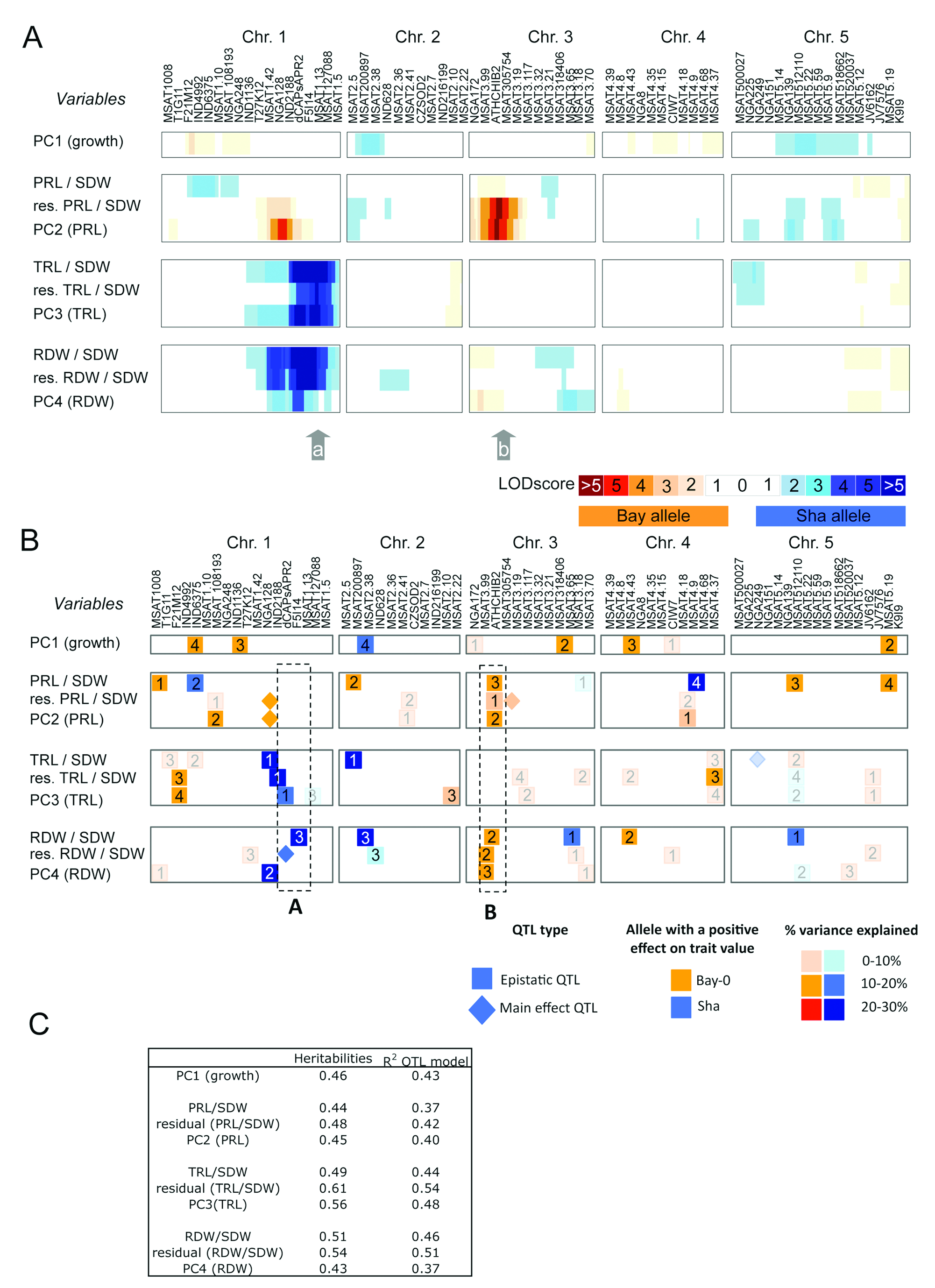

Supplement: Figure S4 — Genetic map of the QTLs detected for root to shoot ratio, residuals of correlations between root variables and shoot dry weight and coordinates in the principal component analysis. Data are those obtained 20 days after sowing. A. Map of the LOD score values all along the genome using Interval Mapping analysis. A color code indicates the parental allele which increases the value of the variables at the marker (blue for Sha alleles, and red for Bay alleles). The LOD score value is shown as different color intensities. Arrows a and b refer to regions described in the text. B. Map of the regions involved in models combining main effects and epistatic QTLs. A color code indicates both the allele which increases the value of the variable at one specific region and the percentage of variance explained by the QTL. Identical numbers are indicated in the two partners of the epistatic interaction. A and B rectangles refer to regions controlling root related variable but not involved in global plant growth. QTLs not retrieved in the map from 24 days after sowing plants are shown with a translucent color. C. Broad-sense heritability and r2 of the QTL models shown in B. (TIF) [file pone.0032319.s004.tif]

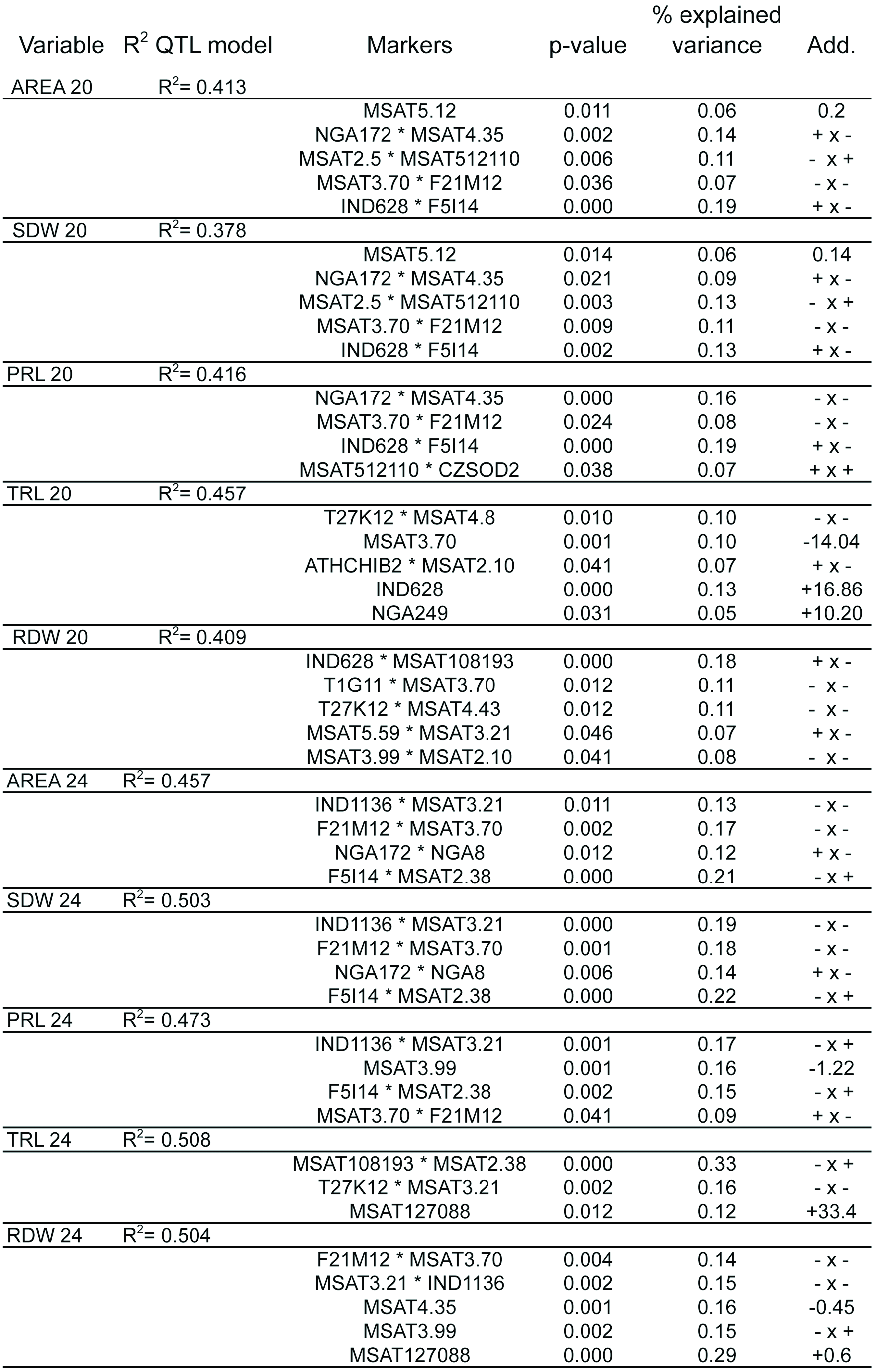

Supplement: Table S1 — QTL models for the shoot and root growth variables. AREA, SDW, PRL, TRL and RDW refer to rosette area, shoot dry weight, primary root length, total root length, and root dry weight respectively. Models are shown for both data at 20 and 24 days after sowing. The percentage of variance explained by the QTL model (R2 QTL model), the markers involved as main effect or epistasic, the p-value of the t-test, the percentage of variance explained by each term of the model, and the corresponding additive effect are indicated. (TIF) [file pone.0032319.s005.tif]

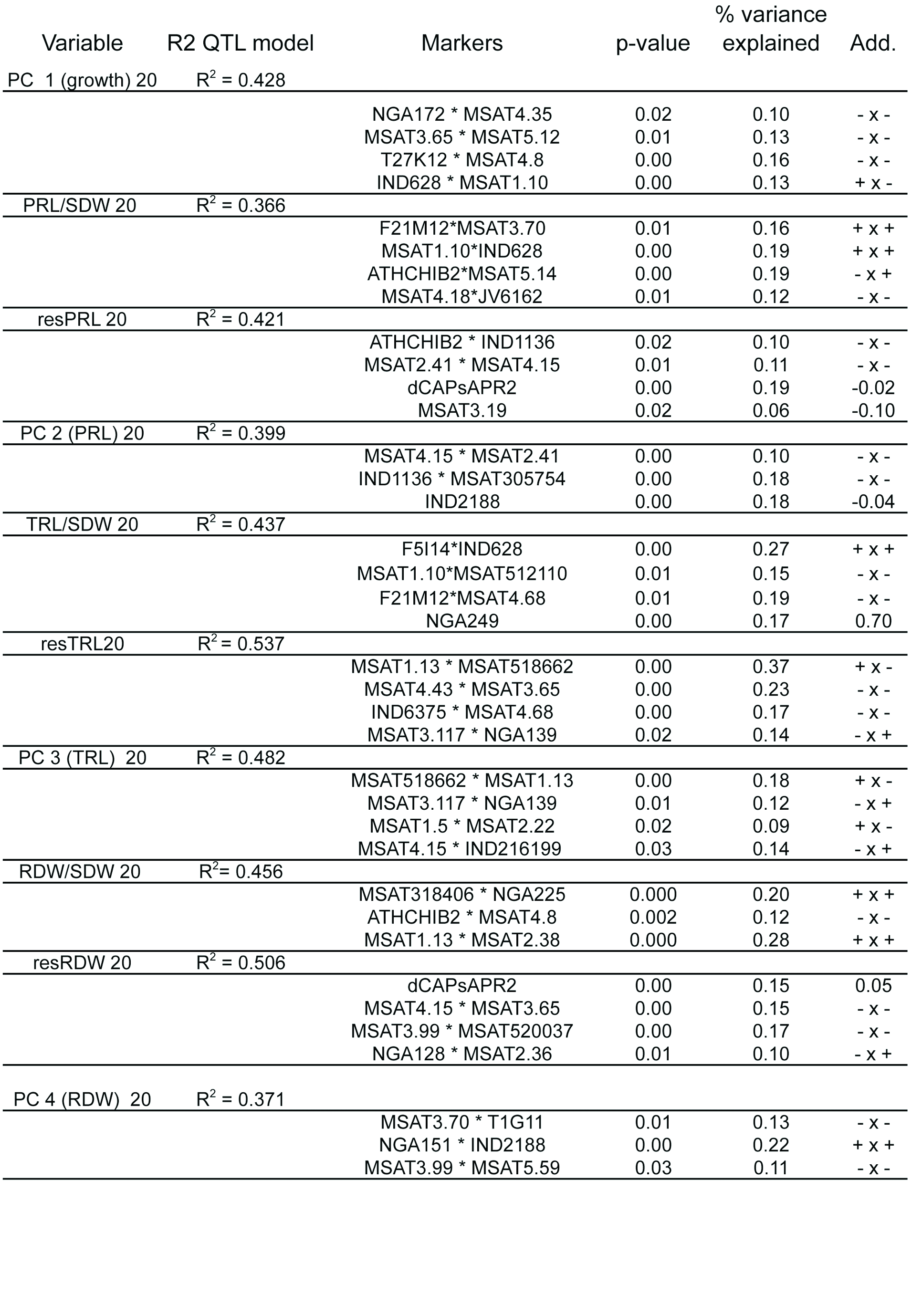

Supplement: Table S2 — QTL models for three types of calculated variables. Ratio between root variables (Root dry weight (RDW), Total root length (TRL), and Primary root length (PRL)) and Shoot dry weight (SDW), PCA coordinates on principal components 2, 3 and 4 (that are accounted for by primary root length, total root length and root dry weight respectively), and residuals of the correlations between root variables and shoot dry weight (SDW), at 20 days after sowing. The percentage of variance explained by the QTL model (R2 QTL model), the markers involved as main effect or epistasic, the p-value of the t-test, the percentage of variance explained by each term of the model, and the corresponding additive effect are indicated. (TIF) [file pone.0032319.s006.tif]

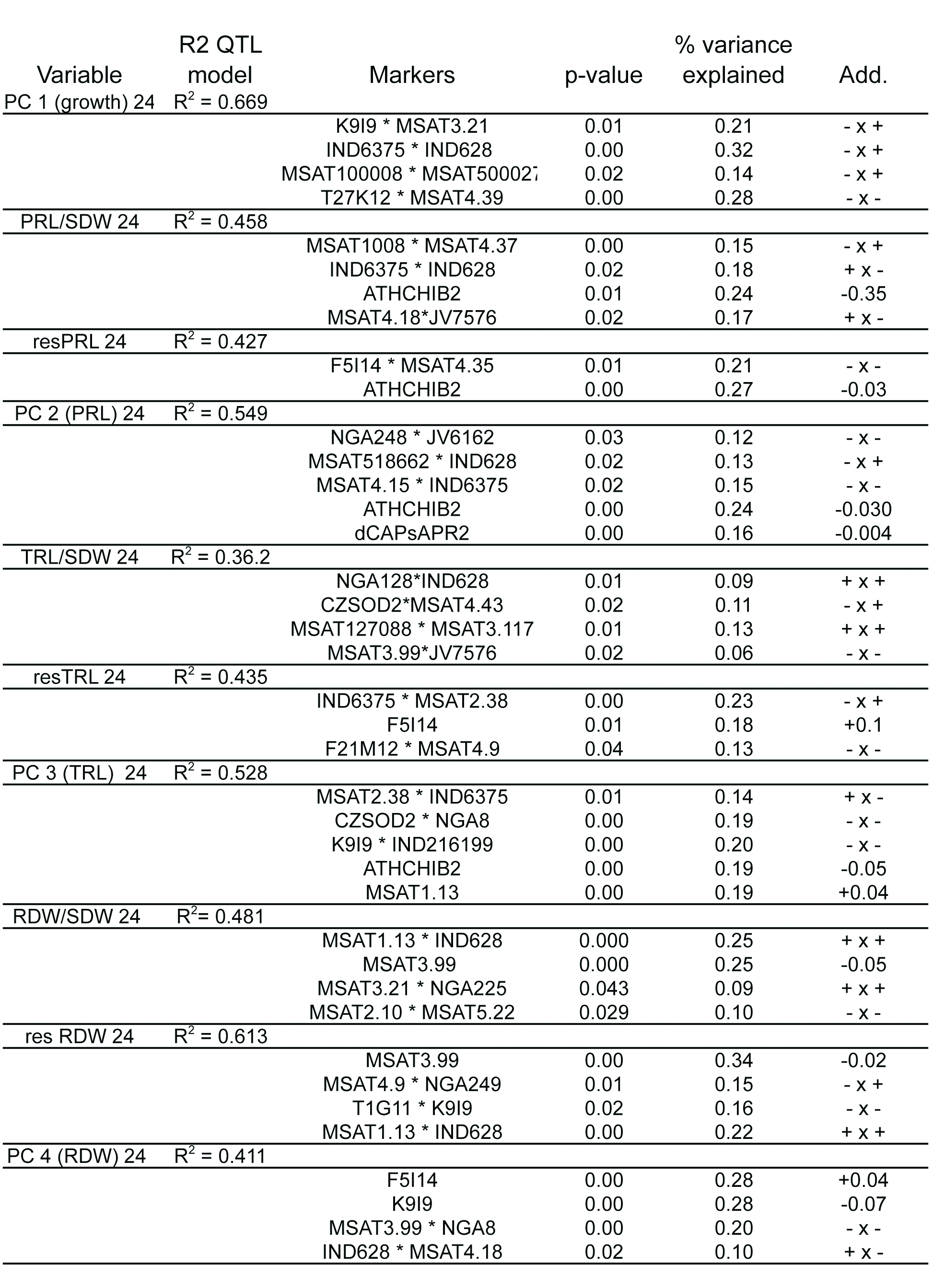

Supplement: Table S3 — QTL models for three types of calculated variables. Ratio between root variables (Root dry weight (RDW), Total root length (TRL), and Primary root length (PRL)) and Shoot dry weight (SDW), PCA coordinates on principal components 2, 3 and 4 (that are accounted for by primary root length, total root length and root dry weight respectively), and residuals of the correlations between root variables and shoot dry weight (SDW), at 24 days after sowing. The percentage of variance explained by the QTL model (R2 QTL model), the markers involved as main effect or epistasic, the p-value of the t-test, the percentage of variance explained by each term of the model, and the corresponding additive effect are indicated. (TIF) [file pone.0032319.s007.tif]

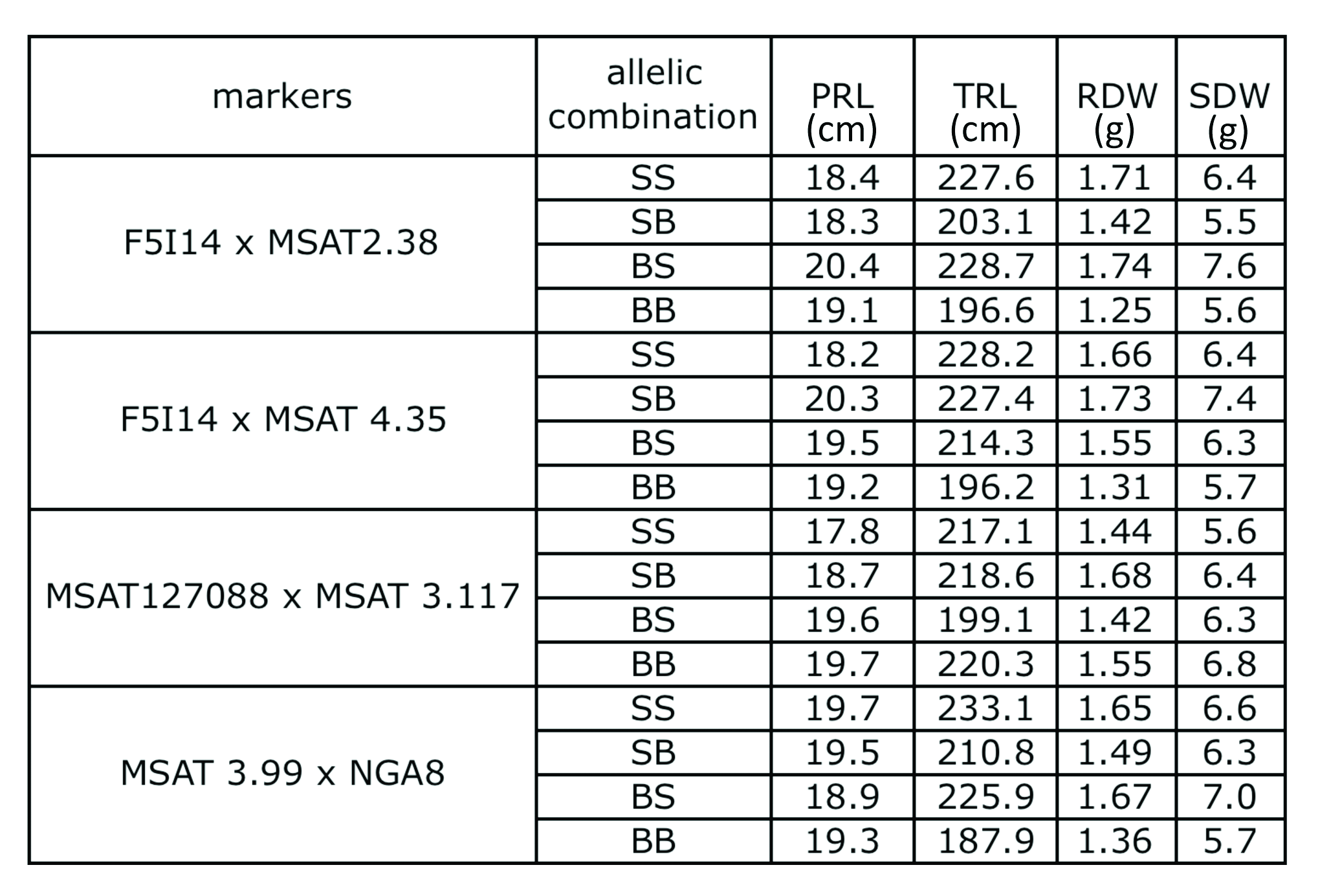

Supplement: Table S4 — Mean (+/−SD) values of root and shoot variables for the RILs in each of the four allelic classes for the 4 epistatic interactions involving the A and B regions: SS, SB, BS and BB refers to the RILs with the Sha or the Bay allele at the first and second marker respectively. (TIF) [file pone.0032319.s008.tif]
